# Systemic Sarcoidosis Mimicking Metastatic Invasive Ductal Carcinoma of the Breast

**DOI:** 10.7759/cureus.87860

**Published:** 2025-07-13

**Authors:** Sai R Vulasala, Christopher D Louviere, Farah Navarro, Grit A Adler, Dheeraj R Gopireddy, Parlyn Hatch, Renato Abu Hana

**Affiliations:** 1 Radiology, University of Florida College of Medicine – Jacksonville, Jacksonville, USA; 2 Pathology and Laboratory Medicine, University of Florida College of Medicine – Jacksonville, Jacksonville, USA

**Keywords:** breast, ct-guided biopsy, invasive ductal carcinoma, metastasis, non-caseating granulomas, pet ct scan, systemic sarcoidosis

## Abstract

Sarcoidosis is a granulomatous inflammatory disorder of uncertain etiology that can closely mimic metastatic malignancies, particularly when it presents with multi-organ involvement. In patients with a confirmed diagnosis of cancer, to avoid misdiagnosis and subsequent inappropriate treatment, distinguishing between sarcoidosis and metastatic disease is essential. Histologic confirmation through tissue sampling and correlation with tumor markers are critical tools in this process. We report a case of a 36-year-old female with invasive ductal carcinoma of the breast who presented with suspicious findings that indicated metastatic disease involving her lungs, liver, and bones. However, tumor marker levels and histopathology revealed systemic sarcoidosis, not metastatic spread.

## Introduction

Granulomatous disorders are characterized by the formation of discrete masses of macrophages and lymphocytes, termed "granulomas," due to autoimmune attacks. Sarcoidosis is an autoimmune granulomatous disorder that can closely mimic metastatic malignancies, particularly when it presents with multi-organ involvement. Some frequently involved sites include the lungs, skin, eyes, and joints. The etiology of sarcoidosis is unknown, and imaging features and clinical symptoms are often nonspecific, including fatigue, weight loss, joint pain, and bone pain [1]. Hilar and mediastinal lymphadenopathy are characteristic imaging findings in patients with sarcoidosis. Sarcoidosis is relatively rare, with less than 250,000 adult individuals affected in the United States, the majority of whom are women [1]. It has a slight predilection for African Americans and individuals of Northern European descent, with an average age of onset of 50 years [1].

Breast cancer is the most common female cancer and the second-leading cause of cancer death in women [2]. In confirmed breast cancer patients, to avoid misdiagnosis and potentially inappropriate treatment, distinguishing between sarcoidosis and metastatic illness is essential. Imaging findings in sarcoidosis and breast cancer may share many similar characteristics and findings, including bone lesions on PET/CT scan or irregular hypoechoic masses on ultrasonography and mammography [1,2]. Therefore, histologic confirmation through tissue sampling and correlation with tumor markers are critical tools in this process. We report a case of a 36-year-old female with invasive ductal carcinoma of the breast who presented with findings that raised suspicions of metastatic disease involving her lungs, liver, and bones. However, tumor marker levels and histopathology revealed systemic sarcoidosis, ruling out metastatic spread.

## Case presentation

**Figure 1:**
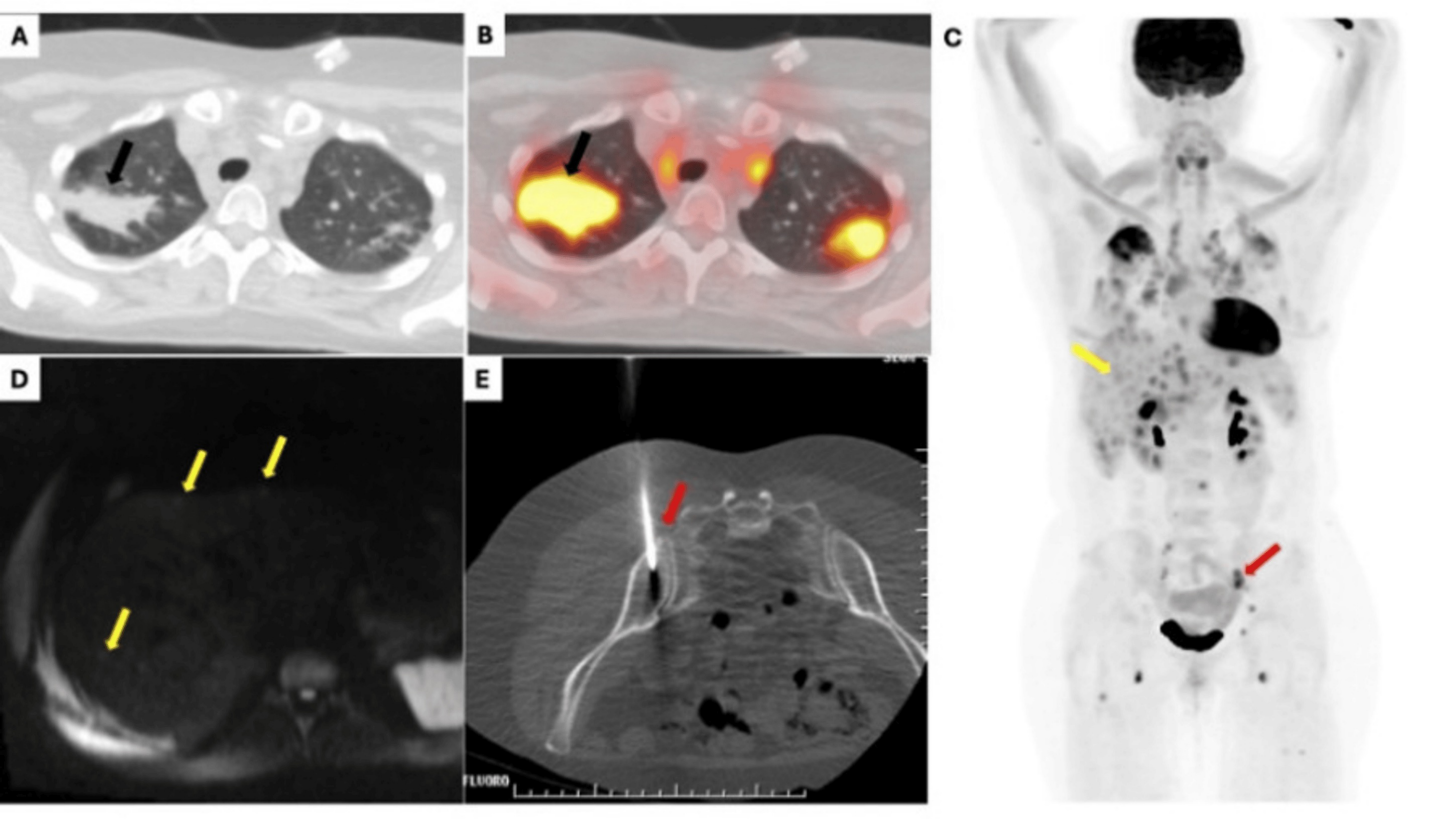
Imaging findings Axial CT (A) of the chest shows consolidative opacity (black arrow) within the right upper lung lobe, which demonstrates high FDG (black arrow) uptake on the corresponding PET (B). PET Maximum intensity projection image (C) demonstrates multiple diffusely distributed foci of FDG avidity in the chest, abdomen, and pelvis. The FDG foci in the liver (yellow arrow) correspond to the foci of diffusion restriction (yellow arrow) on diffusion-weighted images (D). The FDG-avid left iliac lesion (red arrow) was later biopsied for tissue sampling under CT guidance (E) CT: computed tomography; FDG: fluorodeoxyglucose; PET: positron emission tomography

A 36-year-old female with no relevant previous medical history was diagnosed with estrogen receptor-negative and HER2-positive invasive ductal carcinoma of the right breast. A staging PET/CT scan revealed intense 18F-fluorodeoxyglucose (FDG) uptake in the primary breast tumor and liver, duodenum, gastrohepatic ligament region, and right upper lobe of the lung, which raised concern for metastatic disease (Figure 1).

The patient underwent neoadjuvant chemotherapy with TCHP (docetaxel, carboplatin, trastuzumab, and pertuzumab), standard for HER2-positive breast cancer. Follow-up chest CT showed persistent right upper lobe opacities, suggestive of either an inflammatory process or metastasis. A bronchoscopy was performed but revealed no evidence of malignancy. Subsequently, she underwent a bilateral mastectomy with targeted axillary dissection. Surgical pathology revealed no residual tumor in the breast and no axillary lymph node metastases. However, granulomatous inflammation was identified in one of the sentinel lymph nodes.

**Figure 2:**
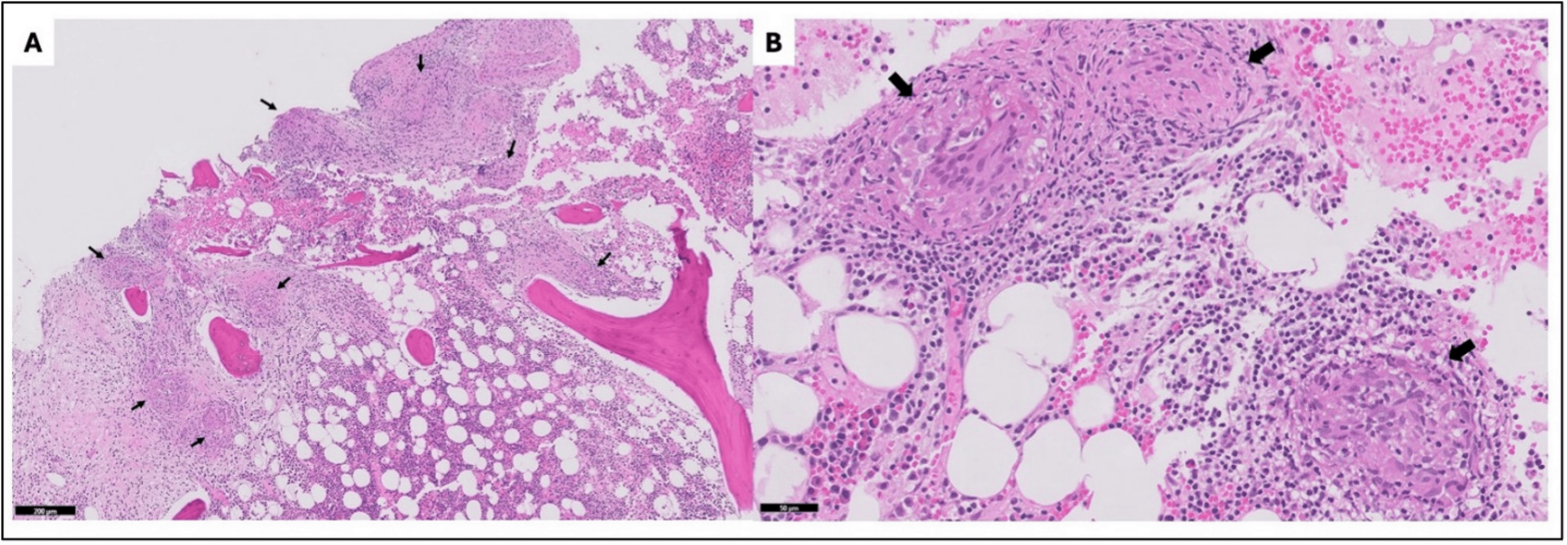
Biopsy and pathology findings (A) Low-power image of the left iliac bone biopsy (H&E stain) shows trabecular bone and bone marrow with multiple granulomas (arrows) surrounded by fibrosis. Scale bar = 200 µm, 5X magnification. (B) High-power image showing well-formed non-necrotizing granulomas (arrows) with surrounding lymphoplasmacytic inflammation, characteristic of sarcoidosis. Pan-cytokeratin, AFB, Fite, and GMS stains (not shown here) were negative. Scale bar = 50 µm, 20X magnification AFB: acid-fast bacillus; GMS: Grocott methenamine silver; H&E: hematoxylin and eosin

Six months later, a follow-up PET/CT demonstrated progression of the right upper lobe consolidation with new bilateral FDG-avid pulmonary micronodules, increased gastrohepatic and porta hepatis lymphadenopathy, and numerous new FDG-avid lesions in the spleen, liver, and bones, including the L3 vertebra, left iliac bone, and right femur (Figure 1). The combined findings escalated suspicion of metastatic disease. Despite these concerning radiographic discoveries, the patient’s CA 15-3 tumor marker levels steadily declined to normal post-treatment. We performed an abdominal MRI to further evaluate the liver lesions, but it yielded inconclusive results (Figure 1). Given the discordance between imaging and tumor markers, we performed a CT-guided biopsy of the left iliac bone (Figure 1). Pathology demonstrated non-caseating granulomas consistent with sarcoidosis (Figure 2).

Based on these findings, we established a diagnosis of systemic sarcoidosis involving the lungs, liver, lymph nodes, and bones. The patient was referred to rheumatology for further management, and continued oncologic surveillance is planned.

## Discussion

Osseous manifestations of sarcoidosis are rare and are seen in only 1-13% of cases, with typical involvement of the small bones of the hand and feet [3]. The involvement of the axial skeleton is infrequent, and very few cases have been reported in the literature [4]. Radiographs may demonstrate honeycomb, lace-like, cyst-like, sclerotic, or radiolucent lesions in the middle and distal phalanges [4]. If involved, the lesions can be lytic, sclerotic, or mixed. Given the rarity of axial skeleton involvement, the primary differential diagnosis to be considered is metastases, especially in patients like ours with a history of cancer. Other most important differentials include osteomyelitis, lymphoma, and multiple myeloma. The absence of monoclonal bands, normal albumin, serum immunoelectrophoresis, and immunoglobulins helps to exclude multiple myeloma. Multimodality imaging and histopathologic evaluation aid in accurate diagnosis and timely clinical intervention.

MRI, nuclear medicine bone scintigraphy, and PET/CT can be utilized for further evaluation and characterization of lytic and bone lesions. Sarcoidosis, metastatic bone disease, and other differential diagnoses share diagnostic imaging findings as described below and pose a clinical challenge in the management of the patient. On MRI, the lytic bone lesions have high T2 and low T1 signal intensity, while the sclerotic bone lesions have low T1 and low to intermediate T2 signal intensity. Both lytic and sclerotic lesions demonstrate intense enhancement on gadolinium administration and mimic metastatic disease [2,5]. Bone scintigraphy scan can be performed with FDG PET/CT or Technitium-99m labeled diphosphonate and helps to identify lesions with high blood flow and high osteoblastic activity. These findings can be seen with osseous sarcoidosis, metastases, stress fractures, and osteomyelitis.

Histopathology shows well-defined non-caseating granulomas, which may demonstrate asteroid and Schaumann bodies [6]. The culture and staining of the tissue sample can help exclude other granulomatous infectious processes such as tuberculosis. Sampling from the axial tuberculosis may often be culture-negative [7]. Sarcoidosis can be differentiated from a sarcoid-like reaction (SLR), which is characteristically devoid of B-cells in the center of the granuloma. Nevertheless, there are cases of SLR devoid of B-cells, which are termed atypical SLR [7]. In this scenario, the predominant nature of T-cells in sarcoidosis helps to accurately diagnose.

The diagnostic imaging findings are nonspecific, making radiographic distinction particularly challenging. Correlation with clinical history and laboratory results is particularly helpful, but histopathology confirmation is often required for definitive diagnosis and management. Our case is similar in this way to previous cases of systemic sarcoidosis masquerading as metastatic malignancy. The lack of tumor marker rise was essential in pivoting diagnostic suspicions away from metastatic malignancy and instead towards alternative diagnoses. Iliac biopsy was essential for correct diagnosis in this case and was chosen due to ease of access and the site's representative bone marrow samples of the entire body.

## Conclusions

Systemic sarcoidosis can closely mimic metastatic disease, especially in oncology patients with FDG-avid lesions across multiple organs. Imaging pitfalls and inadequate diagnostic workup may lead to incorrect and potentially harmful management plans for patients and superfluous emotional suffering. Our report highlights the importance of evaluating multiple differential diagnoses and the crucial role of a multidisciplinary approach with diagnostic imaging and histopathology in clinical decision-making. In cases of potentially metastatic disease with discordant imaging and tumor marker findings, clinicians must consider alternative and seemingly deceiving pathologies.
